# Man with Massively Enlarged Scrotum

**DOI:** 10.1016/j.acepjo.2026.100497

**Published:** 2026-08-22

**Authors:** Alex Lee, Candice Frencl Mateja

**Affiliations:** 1 Department of Internal Medicine, AdventHealth Orlando, Orlando, Florida, USA; 2 Internal Medicine, Morsani College of Medicine, University of South Florida, Tampa, Florida, USA

**Keywords:** cirrhosis, ascites, scrotum, hepatology, urology

## 1 Patient Presentation

**Figure 1:**
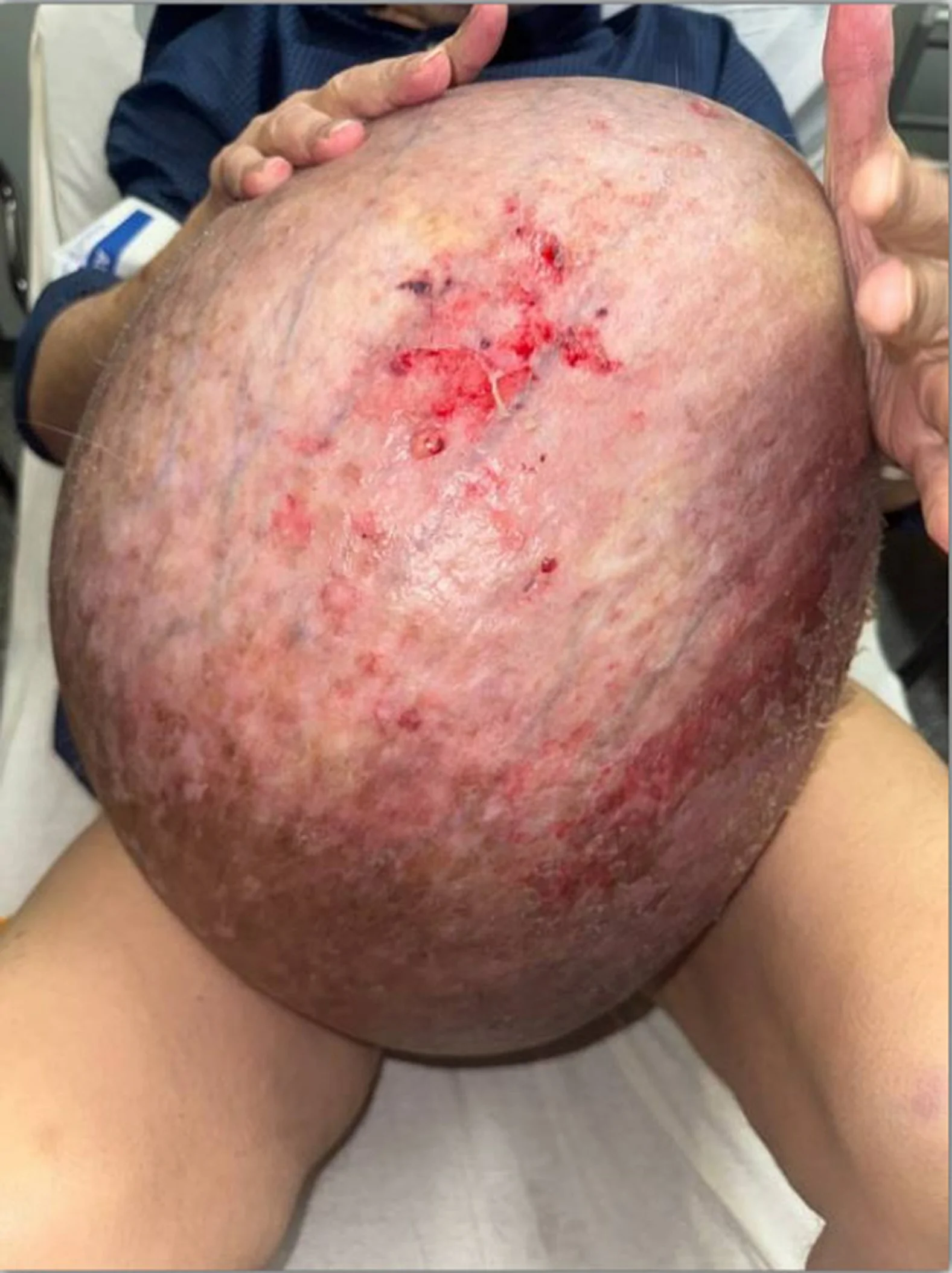
Significantly enlarged scrotum. The scrotal skin appeared maroon and tender and exhibited nonblanchable erythema, consistent with friction-related skin breakdown. Multiple wounds and areas of redness with some secretions were noted. The penis and urethral meatus were not visible due to burial within the massively enlarged scrotum.

A 67-year-old man presented with 3 days of scrotal pain, erythema, and drainage. His history included repaired aortic dissection, urinary tract infection, hypertension, dyslipidemia, benign prostatic hypertrophy, and a longstanding inguinal hernia. Examination showed a markedly enlarged scrotum (Fig 1), with maroon, tender, nonblanchable skin, and draining wounds. No abdominal distension or evident ascites was noted. Laboratory studies were significant for leukocytosis, mild transaminitis, and a high hepatitis C viral load. Computed tomography scan revealed a large, fluid-filled sac within the scrotum, originating from an inguinal hernia and hydrocele but primarily composed of a significant ascitic fluid. The testicles were not involved. Imaging also showed cirrhosis with portal hypertension. Notably, the scrotal sac was predominantly filled with ascitic fluid, while no ascites was detected in the abdominal cavity.

## 2 Diagnosis: Massive Scrotal Ascites as a Presentation of Cirrhosis

**Figure 2:**
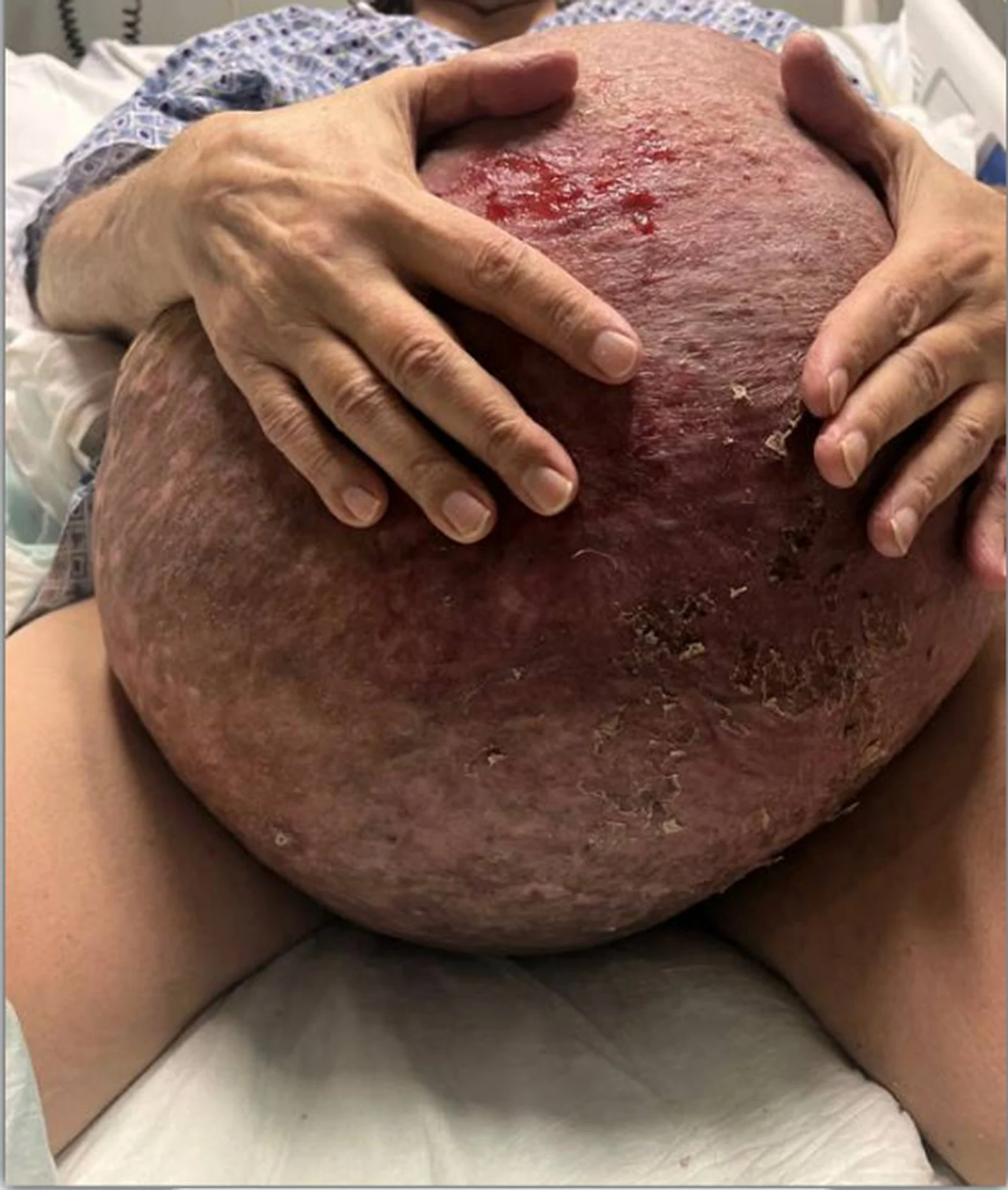
To relieve tension in the scrotum, prevent further skin breakdown, and reduce the risk of perforation or severe infection, a real-time ultrasound-guided scrotal centesis was performed. Using a 5-French Yueh needle and pigtail catheter connected to a vacuum bottle suction system, 9.4 L of clear yellow fluid were aspirated from the peritoneal space. Following the procedure, the scrotum remained severely enlarged, but the size and tension of scrotal skin were significantly reduced.

For inguinal hernia and cirrhosis with massive ascitic fluid collection in scrotum, there was concern for infection and progressive skin compromise, so ultrasound-guided scrotal centesis was performed.^1^ The patient was managed with diuretics, topical antimicrobials, and wound care, with significant reduction in scrotal size (Fig 2).

The images demonstrate an unusual presentation of cirrhosis with isolated massive scrotal ascites in the absence of clinically apparent intra-abdominal fluid.^2^ Chronic inguinal hernia anatomy likely facilitated dependent fluid tracking, resulting in a misleading presentation of portal hypertension. In the emergency department, this presentation can mimic a urologic emergency and prompt urgent surgical intervention; recognizing the underlying cirrhosis and dependent ascitic fluid tracking instead redirects management toward drainage, ascites control, and wound care, averting avoidable harm.^3^

## Funding and Support

By *JACEP Open* policy, all authors are required to disclose any and all commercial, financial, and other relationships in any way related to the subject of this article as per ICMJE conflict of interest guidelines (see www.icmje.org). The authors have stated that no such relationships exist.

## Conflict of Interest

All authors have affirmed they have no conflicts of interest to declare.
